# Supplementary material for: Low-Cost, Scalable Simulations in Obstetric Trauma and Resuscitative Hysterotomy for Emergency Medicine Residents
Source: MedEdPORTAL. 2024 Oct 3;20:11452. doi: 10.15766/mep_2374-8265.11452 (PMC11447011; doi:10.15766/mep_2374-8265.11452)
Supplement: Supplementary file 1 — List of Required Equipment.docxResuscitative Hysterotomy Task Trainer Construction.docxSimulation Case.docxQuestionnaire.docx [file mep_2374-8265.11452-s001.zip › C. Simulation Case.docx]

This appendix presents the details of the simulation case. Please refer to this appendix when prompted in the main text for a more in-depth understanding of the scenario. Each section is clearly labeled for ease of reference. In the section on “simulation case flow”, the case events are presented chronologically.

| **Appendix C: Simulation Case**    **SIMULATION CASE TITLE:** Obstetric Trauma & Resuscitative Hysterotomy  **AUTHORS:** Dr. Chin Hao Ren, Dr. Ng Wei Xiang  **LEARNER AUDIENCE:** Emergency Medicine Resident | |
| --- | --- |
| **PATIENT NAME:** Mdm Lim  **PATIENT AGE:** 30 years old female  **CHIEF COMPLAINT:** Victim of road traffic accident, with hypotension  **PHYSICAL SETTING:** Tan Tock Seng Hospital (TTSH) Simulation and Integrated Medical Training Advancement Centre (SIMTAC) Emergency Room | |
| **Brief Narrative Description of Case** | This is a scenario on trauma resuscitation of a young pregnant female who had hypotension after being involved in a road traffic accident. The patient subsequently went into cardiac arrest and required resuscitative hysterotomy. Residents will work as a team to resuscitate the patient and perform resuscitative hysterotomy. |
| **Primary Learning Objectives** | 1. Demonstrate systematic, coordinated resuscitation based on Advanced Trauma Life Support (ATLS) principles. 2. Display leadership skills by demonstrating excellent teamwork, manpower management, situational awareness, and closed-loop communication. 3. Recognize maternal anatomical, physiological changes due to pregnancy, their implications to resuscitation. 4. Demonstrate knowledge of special considerations in trauma resuscitation of a pregnant patient, such as estimating gestational age/ viability, radiological imaging, Rhogam administration, fetal monitoring, etc. 5. Demonstrate resuscitative hysterotomy. |
| **Critical Actions** | 1. Assess patient following Advanced Trauma Life Support (ATLS) protocols, which includes primary survey, use of adjunct and investigations. 2. Identify breathing (tension pneumothorax) and circulation (hemorrhagic shock from uterine rupture) issues. 3. Institute hemodynamic resuscitation, including lateral displacement of the uterus, crystalloid infusion, blood transfusion, tranexamic acid, and relief of tension pneumothorax. 4. Recognize deterioration of patient into cardiac arrest and provide appropriate resuscitation according to Advanced Cardiac Life Support (ACLS) protocols, which includes CPR, intubation and use of ACLS drugs. 5. Recognize the need for resuscitative hysterotomy and demonstrate the steps of resuscitative hysterotomy. |
| **Learner Preparation or Prework** | Pre-reading materials^7^ (disseminated to residents 1 week before the session). |

**Simulation Case Flow**

| **Time** | **Event / Simulated event** | **Faculty’s actions / Cues (in italics)** | **Mannequin’s description** | **Targeted learner response** |
| --- | --- | --- | --- | --- |
| 0-1 min | Preparation | Broadcast message:  Standby for a 30-year-old female, victim of road traffic accident with hypotension  *Slide 1: Standby message* | Patient (manikin) lies on trolley | - Prepare the resuscitation team for the patient’s arrival:  - Assignment of roles - Appropriate personal protection equipment - Equipment e.g.: airway, ultrasound, pelvic binder etc. - Consider prehospital trauma activation |
| 1-3 min | Arrival  30-year-old female  Ms Lim  Pushed in by SCDF paramedics | Informs learners of patient’s arrival  Paramedic’s report:  The patient was a pedestrian walking across a T-junction, hit by a car traveling at 40-50 km/hr, flung 3 meters. Non-ambulatory at the scene.  GCS E3V4M5, BP 90/60, pulse 100 en route. Groaning in pain  Unsure of past medical history or pregnancy status  No known allergies  No visible blood on the scene (only volunteer this information if asked by learner) | Patient in c-collar & on spinal board  T 37 degree Celsius  BP 80/60 mmHg  HR 120 / min  SpO2 on room air 92%  GCS E3V3M5  Moaning in pain over chest and abdomen | - Learners receive paramedic’s report  - Trauma activate based on mechanism + vitals |
| 3–5 mins | Primary survey | Provide primary survey information  *Slide 2: FAST demonstrating presence of free fluid, extrauterine hematoma, fetal heartbeat present*  *Slide 3: Absent lung sliding/ barcode signs on right lung*  *Slide 4: Chest X-ray: right tension PTX (if done)*  *Slide 5: Pelvis X-ray: no fracture* | Primary survey  A: own airway, no blood/ FB  B: SPO2 92% RA, right-sided AE reduced, right sided chest compression positive  C: BP 80/60 HR 120. Tender uterus just above umbilicus, palpable fetal parts, ecchymosis over abdomen, vaginal bleeding  Pelvic compression negative  D: pupils 2 mm equal reactive, E3V4M5. Moaning in pain over the right chest and abdomen. No external signs of head injury  E: no limbs' deformity/ swelling/ bleeding  Adjunct  FAST: presence of free fluid, right lung sliding absent, extrauterine hematoma  Chest X-Ray: right tension pneumothorax  Pelvis X-ray: no fracture  Log roll  No back injury  Digital rectal examination: anal tone intact, no blood | - Primary survey: ABCDE  - Obtain AMPLE (Allergy, Medication, Past medical history, Last meal, Event) history  - Put on monitoring devices  - Point of care test: Hypocount, ECG, urine pregnancy test, blood gas with lactate  - Labs: FBC, UECr, PT/PTT, GXM, Kleihauer-Betke test (KB) test  - Fetal HR monitoring  - Activate Trauma and O&G teams  - Learners note that the patient has B/C problem i.e. Right pneumothorax, & hemorrhagic shock likely from uterine rupture |
| 5–10 mins | Management | Learners are expected to demonstrate the steps of chest tube insertion (including the level of entry) | BP 60/40  HR 135  Post chest tube placement: gush of air, no blood, underwater seal oscillating, SpO2 improves to 97%  However, persistent hypotension, getting drowsy, GCS E1, V2, M5, pupils 2mm2mm reactive, no lateralizing signs | - Oxygen supplementation  - Lateral displacement of uterus/ left lateral tilt  - IV crystalloid  - Activate E-blood if not already, consider massive transfusion protocol (ABC score: 3)  - IV tranexamic acid 1g  - IM/ IV Rhogam 30mls if Rhesus (-)  - Demonstrate the steps of right chest tube thoracostomy (insert chest tube 2 or more ICS above)  - Pain relief |
| 10–15 mins | ACLS resuscitation | Informs that patient not responsive | Unrecordable vitals  Not responsive  No pulse  Rhythm: PEA | - Reassess patient and discover deterioration to PEA cardiac arrest  - Demonstrate resuscitation based on ACLS & ATLS principles  - bilateral thoracostomy  - High-quality CPR  - Definitive airway via endotracheal intubation  - Adrenaline 1 mg 1:10,000 every 3-5mins |
| 15–20 mins | Resuscitative Hysterotomy | Inform learners:  4 mins up, pulse check still PEA collapse.  *Learners describe the steps of these procedures.*  *Learners will get an opportunity to go through resuscitative hysterotomy via simulation model* | Unrecordable vitals  Not responsive  No pulse  Rhythm: PEA | - Describes the indications and steps of resuscitative hysterotomy  - Demonstrate the steps of resuscitative hysterotomy via simulation model  - Prepare another team for neonatal resuscitation  - prepare Children’s Hospital Emergency Transport Service (CHETS) if applicable |
| 20–25 mins | Scenario ends  Summary and Debrief | Inform learners:  - Patient ROSC post resuscitative hysterotomy  - Trauma team decides to send patient to Operating Theatre as P0 case for exploratory laparotomy  - Scenario ends | ROSC  BP 90/60  HR 140  SpO2 100 % | - Learners will clarify doubts/asks questions if any |

*Acronym: BP (blood pressure), HR (heart rate), SPO2 (capillary oxygen saturation), T (temperature), GCS (Glasgow Coma Scale), FAST (Focused Assessment With Sonography for Trauma)

**Ideal Scenario Flow**

The learners enter the room and receive the standby message over the intercom. They prepare to receive the patient by assigning roles, donning personal protection equipment, and preparing equipment such as advanced airway devices, ultrasound machines, pelvic binder etc. Once the patient arrives, learners receive a handover from the paramedic and take an AMPLE history. Learners make the decision to proceed with trauma activation in view of the mechanism of injury and unstable vital signs. Learners perform a primary survey on the patient, which reveals a right pneumothorax and hemorrhagic shock from uterine rupture. Learners institute appropriate measures such as oxygen supplementation, lateral displacement of uterus, crystalloid infusion, blood transfusion, tranexamic acid, and right chest tube insertion. The patient continues to deteriorate into PEA cardiac arrest. Learners resuscitate the patient as per ACLS, which includes high quality CPR, endotracheal intubation, and ACLS drugs. They also perform bilateral thoracostomy. The patient remains in PEA after 4 minutes of resuscitation, and the learners perform resuscitative hysterotomy on the simulation model. The patient has ROSC after the resuscitative hysterotomy. The scenario ends after handing the patient and the neonate over to the trauma team and the neonatology team respectively. The scenario lasts for approximately 20 minutes.

**Anticipated Challenges**

Unfamiliar with steps of resuscitative hysterotomy: Residents may be unfamiliar with the steps of resuscitative hysterotomy and find themselves unable to proceed with the scenario. We aim to mitigate this with:

1. Sending pre-reading materials to the residents 1 week prior to the session
2. Real-time guidance by faculty during the scenario. Faculty facilitating the scenario will step in if the residents are unable to proceed and guide the learners step-by-step.
