# Supplementary material for: Low-Cost, Scalable Simulations in Obstetric Trauma and Resuscitative Hysterotomy for Emergency Medicine Residents
Source: MedEdPORTAL. 2024 Oct 3;20:11452. doi: 10.15766/mep_2374-8265.11452 (PMC11447011; doi:10.15766/mep_2374-8265.11452)
Supplement: Supplementary file 1 — List of Required Equipment.docxResuscitative Hysterotomy Task Trainer Construction.docxSimulation Case.docxQuestionnaire.docx [file mep_2374-8265.11452-s001.zip › D. Questionnaire.docx]

**Feedback on: Obstetric Trauma Simulation**

Dear all,

You have been invited to participate in this survey as you participated in the Obstetric Trauma Simulation. We would like your valuable feedback to improve on future sessions, and we hope to publish our experience in a medical/education journal. This survey will take approximately 5 minutes to complete.

Your decision to take part in this survey is entirely voluntary. There are no risks to individuals participating in this survey.

By filling out this form, you are giving us implied consent to use your feedback in related studies. If you do not wish to complete the survey, you may close your browser.

Thank you in advance for your active participation and valuable feedback! Please contact us directly if you have any questions related to the survey.

Regards,

Dr Chin Hao Ren

Dr Ng Wei Xiang

**Relevance of Training**

Question 1: Was the obstetric trauma simulation scenario relevant to my job?

- 1 (Not relevant at all)
- 2
- 3
- 4
- 5 (Highly relevant)

Question 2: Was the complexity of the obstetric trauma simulation appropriate for my level of training?

- Too simple
- Slightly too simple
- Just about right
- Slightly too complicated
- Too complicated

Question 3: Did the obstetric trauma simulation help in translating my textbook knowledge to clinical skills?

- 1 (Not at all)
- 2
- 3
- 4
- 5 (Yes, very much)

**Task Trainer Specific**

Question 4: Have you performed / assisted / observed a cesarean section previously?

- Yes
- No

Question 5: How would you rate the realism of the task trainer?

- 1 (Does not resemble anything at all)
- 2
- 3
- 4
- 5 (As real as it could practically be)

Question 6: Are you clearer about the steps of performing a resuscitative hysterotomy after the session?

- 1 (Much more confused)
- 2
- 3
- 4
- 5 (Very much clearer)

Question 7: Did you feel more confident in performing or assisting in a resuscitative hysterotomy after the session?

- 1 (I feel much less confident)
- 2
- 3
- 4
- 5 (I feel much more confident)

**Overall Rating**

Question 8: Did the simulation increase your confidence of dealing with a similar clinical scenario?

- 1 (I feel much less confident)
- 2
- 3
- 4
- 5 (I feel much more confident)

Question 9: How keen are you to participate in a similar training/ simulation session?

- 1 (Not keen at all)
- 2
- 3
- 4
- 5 (Count me in every time!)

Question 10: Please let us know of any comments or suggestions for improvement.

_____________________________________________________________________________________________

_____________________________________________________________________________________________

_____________________________________________________________________________________________
