# Supplementary material for: Low-Cost, Scalable Simulations in Obstetric Trauma and Resuscitative Hysterotomy for Emergency Medicine Residents
Source: MedEdPORTAL. 2024 Oct 3;20:11452. doi: 10.15766/mep_2374-8265.11452 (PMC11447011; doi:10.15766/mep_2374-8265.11452)
Supplement: Supplementary file 1 — List of Required Equipment.docxResuscitative Hysterotomy Task Trainer Construction.docxSimulation Case.docxQuestionnaire.docx [file mep_2374-8265.11452-s001.zip › A. List of Required Equipment.docx]

**Appendix A: List of Required Equipment**

Equipment:

- 1 female mannikin: with made-up bruising over abdomen
- 1 resuscitative hysterotomy task trainer (refer to Appendix B) and equipment: scalpel, scissors, artery clamp x 2, retractor x 2
- 1 chest tube task trainer and equipment: scalpel, artery clamp, chest tubes, silk suture, underwater seal
- 1 bed/trolley
- Blood pressure monitor, 3-lead ECG with pulse oximetry
- Oxygen delivery devices: nasal prongs, face mask, venturi mask, non-rebreather mask, bag-valve-mask
- Airway equipment: Direct/video laryngoscope, bougie, various sizes of endotracheal tubes, laryngeal mask airways
- Defibrillator
- Ultrasound machine
- Cervical collar
- Spinal board
- T-pod device
- Large bore IV cannula taped over patient’s antecubital fossa
- Hypocount machine, blood tubes
- 1 unit 0.9% normal saline solution
- 1 unit “blood”
- IV tubing
- Computer with projection to display PowerPoint slides
