# Supplementary material for: Low-Cost, Scalable Simulations in Obstetric Trauma and Resuscitative Hysterotomy for Emergency Medicine Residents
Source: MedEdPORTAL. 2024 Oct 3;20:11452. doi: 10.15766/mep_2374-8265.11452 (PMC11447011; doi:10.15766/mep_2374-8265.11452)
Supplement: Supplementary file 1 — List of Required Equipment.docxResuscitative Hysterotomy Task Trainer Construction.docxSimulation Case.docxQuestionnaire.docx [file mep_2374-8265.11452-s001.zip › B. Resuscitative Hysterotomy Task Trainer Construction.docx]

Appendix B: Construction of Resuscitative Hysterotomy Task Trainer

| 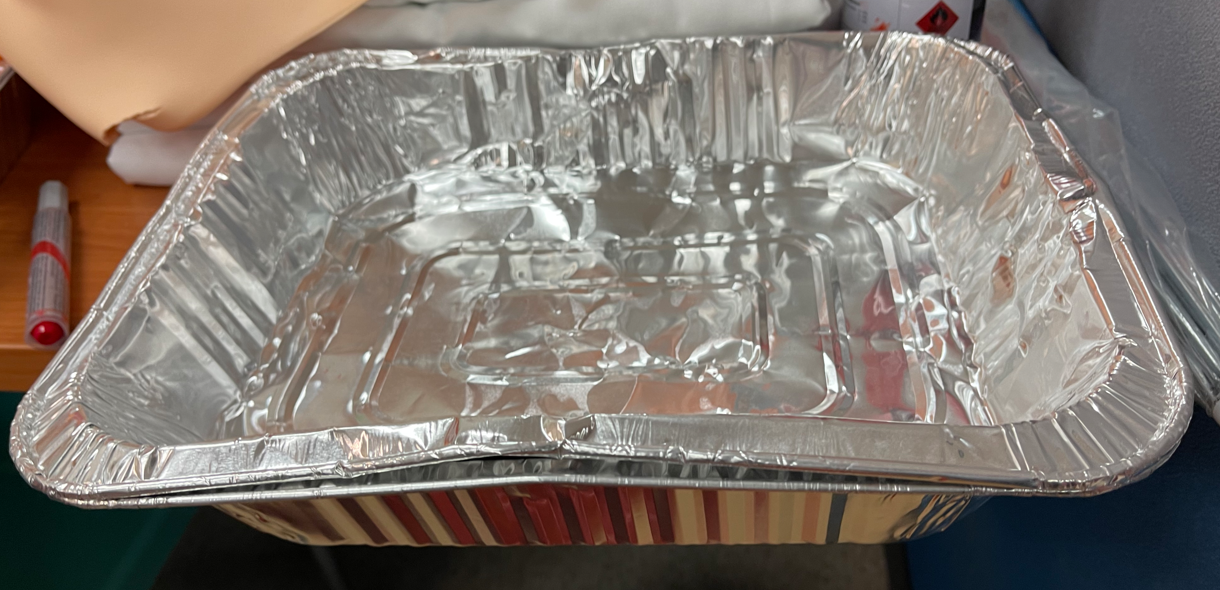 | Step 1: Two aluminum trays were stacked together to form base support |
| --- | --- |
| 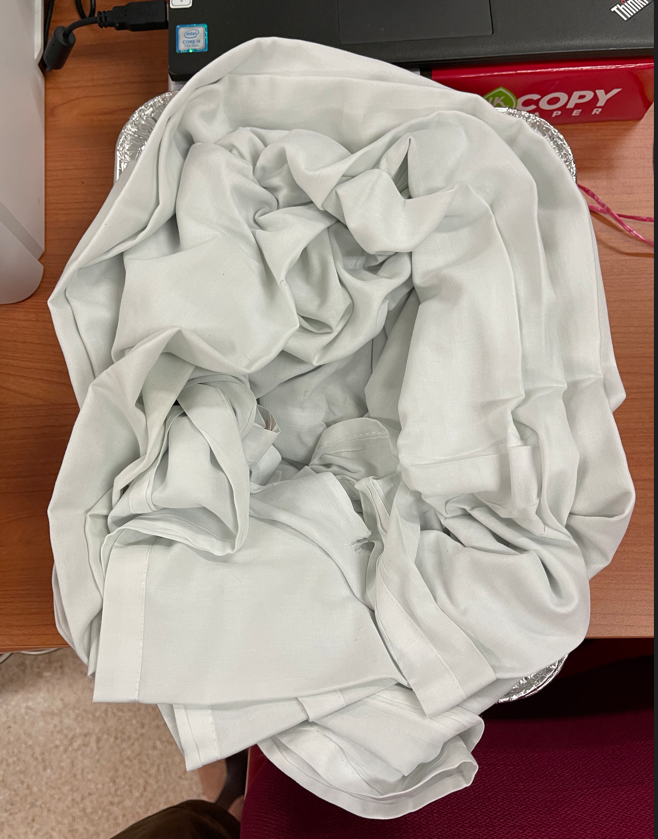 | Step 2: A bedsheet was folded and placed in the aluminum trays to resemble “intra-abdominal organs” |
| 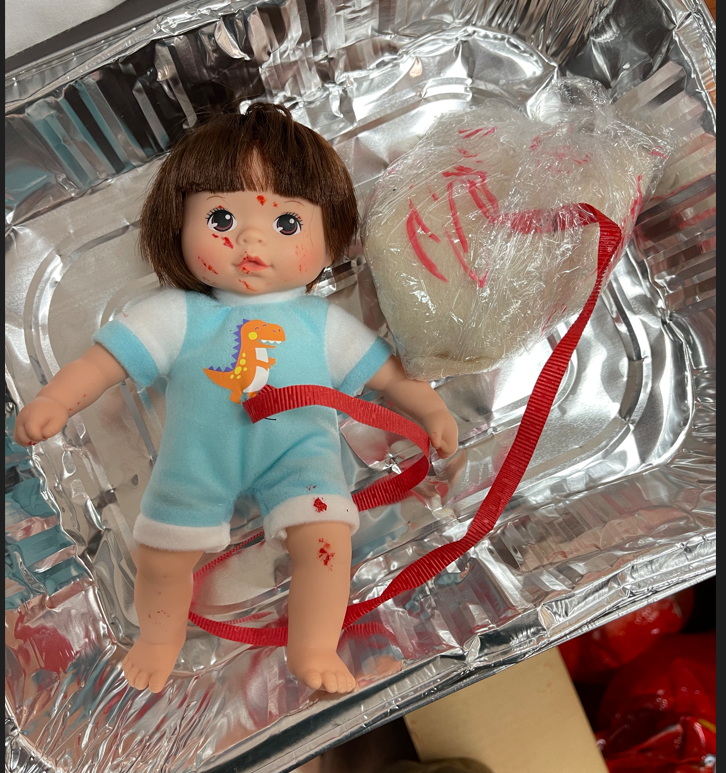 | Step 3: A red-colored string was attached to a “baby” doll to resemble an “umbilical cord”, while the other end of the string was attached to a crumpled kitchen cling wrap, resembling a “placenta” |
| 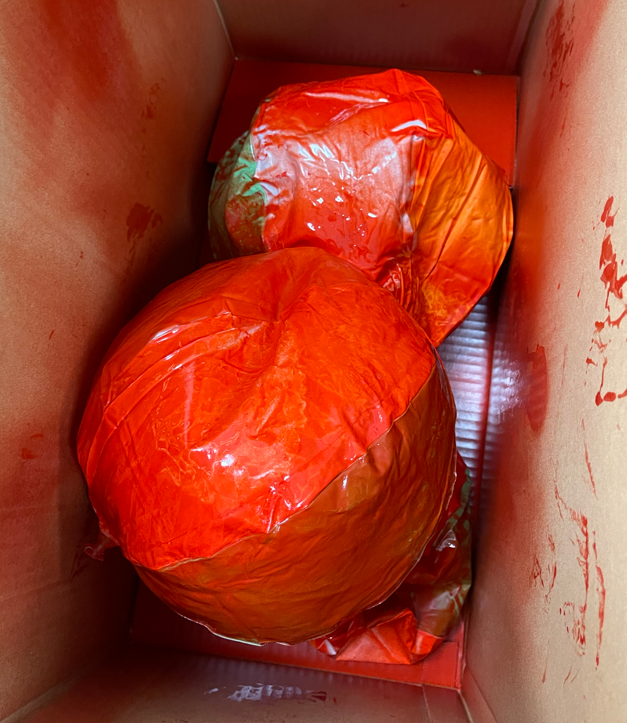 | Step 4: A 40 cm beach ball was painted red to resemble a “uterus”. The top of the beach ball was cut to create a small opening |
| 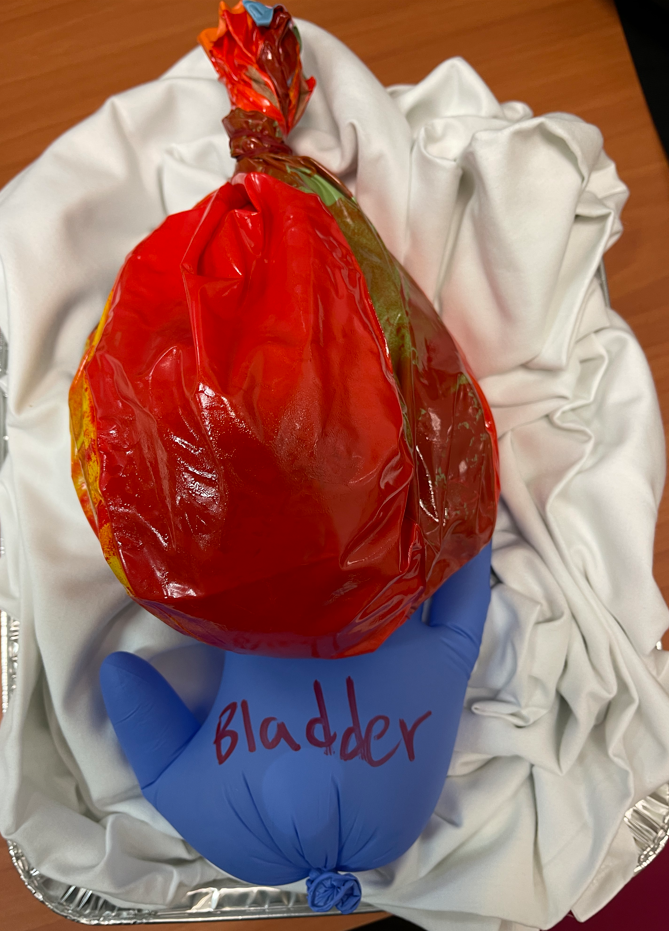 | Step 5: The “baby”, “umbilical cord”, “placenta” and a water-filled balloon (resembling amniotic fluid sac) were packed into the beach ball. The open end of the beach ball was wrapped with a rubber band. The wrapped beach ball was placed on top of the folded bedsheet. A water-filled glove was placed just below the “uterus” to resemble the “bladder” |
| 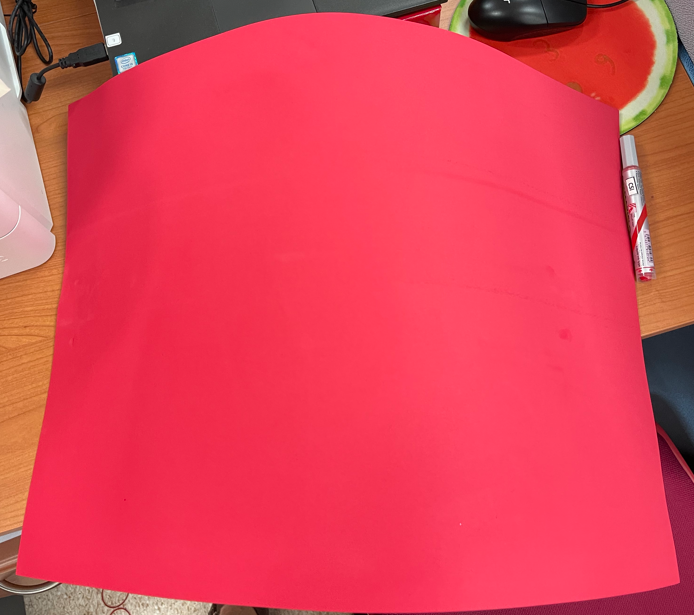 | Step 6: One layer of red foam sheet was placed to resemble “abdominal muscle” |
| 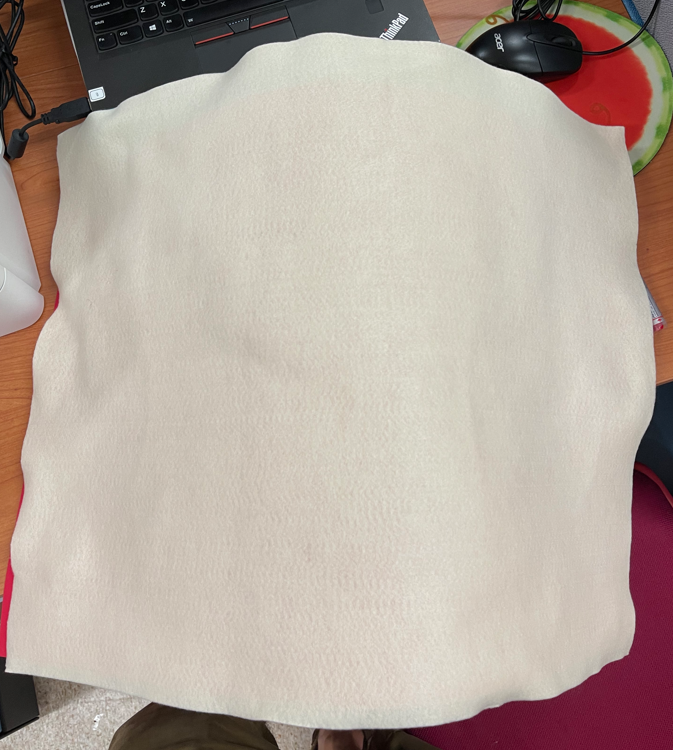 | Step 7: One layer of cream felt cloth was placed to resemble “subcutaneous tissue” |
| 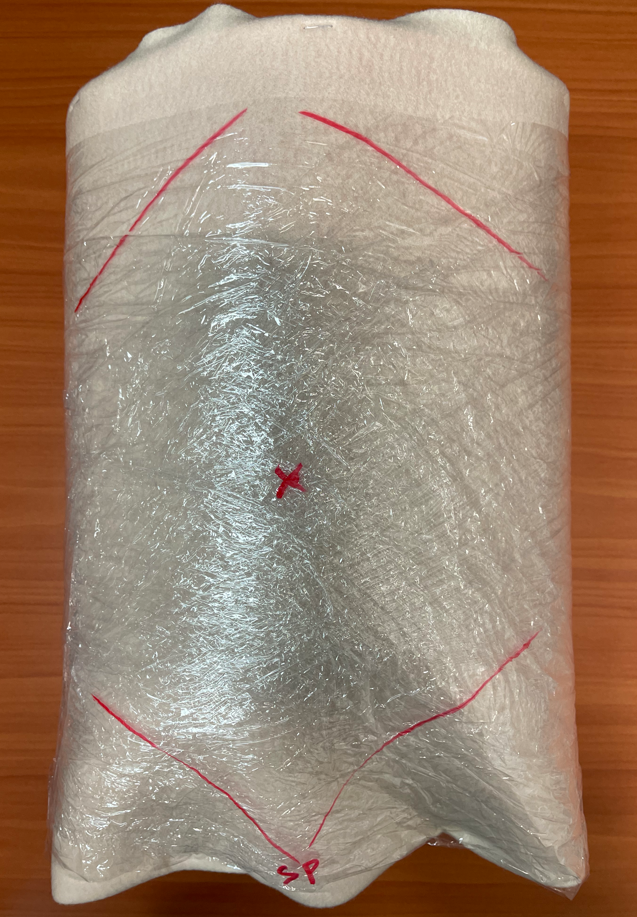 | Step 8: The entire model was wrapped with a few layers of kitchen cling wrap to resemble “skin”. Landmarks were drawn using a marker pen on the kitchen wrap to indicate subcostal margin, umbilicus and inguinal crease |
| 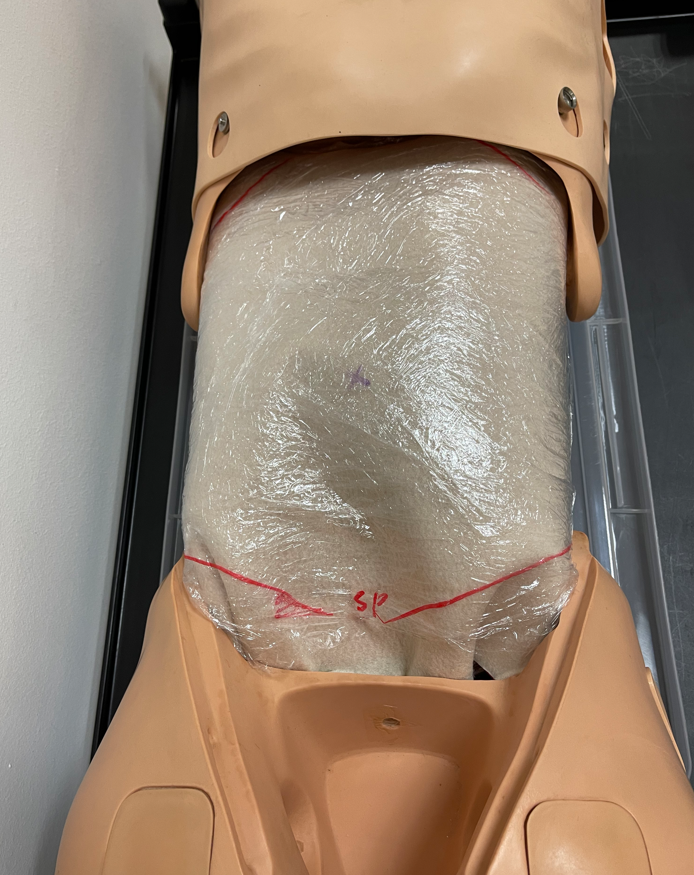 | Step 9: The model was assembled with human manikin (without the abdomen component) as a resuscitative hysterotomy task trainer |
