# Supplementary material for: Low-Cost, Scalable Simulations in Obstetric Trauma and Resuscitative Hysterotomy for Emergency Medicine Residents
Source: MedEdPORTAL. 2024 Oct 3;20:11452. doi: 10.15766/mep_2374-8265.11452 (PMC11447011; doi:10.15766/mep_2374-8265.11452)
Supplement: Supplementary file 1 — List of Required Equipment.docxResuscitative Hysterotomy Task Trainer Construction.docxSimulation Case.docxQuestionnaire.docx [file mep_2374-8265.11452-s001.zip › _Educational Summary Report_11452.pdf]

MedEdPORTAL® The AAMC Journal of Teaching and Learning Resources

Original Publication Open Access

Low-Cost, Scalable Simulations in Obstetric Trauma and Resuscitative Hysterotomy for Emergency Medicine Residents

Hao Ren Chin, MBBS, MRCEM*,†, Wei Xiang Ng, MBBS, MMed (Emergency)†

*Corresponding author: haoren_chin@ttsh.com.sg
†Co-primary author

Abstract

Introduction: Simulation-based learning is essential for health care providers to prepare for rare obstetric emergencies, such as severe trauma and maternal cardiac arrest. These situations demand rapid and prompt actions, often testing the skill of emergency physicians. Resuscitative hysterotomy (RH), a critical procedure in maternal cardiac arrest, requires technical expertise, coordination, and anatomical knowledge. The high cost of commercial trainers and complex existing models restricts accessibility. This resource introduces a low-cost anatomically accurate RH task trainer and assesses its effectiveness in improving skills and confidence among trainee emergency physicians. **Methods:** A 20-minute-long case scenario depicted the resuscitation of a pregnant trauma patient with tension pneumothorax and uterine rupture, culminating in maternal cardiac arrest necessitating RH. Residents performed RH on the task trainer under faculty guidance. Feedback followed the Pendleton model, and an online questionnaire gauged the residents' experiences. **Results:** Thirty emergency medicine residents participated in the simulation. The questionnaire revealed positive responses, confirming the session's relevance and enhancement of clinical skills and confidence. **Discussion:** Our results underscore the RH task trainer's critical role in improving residents' skills and confidence during obstetric trauma simulations. Its realism and effectiveness were notably well received. Future refinements aim to augment fidelity while preserving affordability and integrating regular reinforcement sessions. This innovative educational approach equips health care professionals to respond adeptly to rare and challenging obstetric emergencies, ultimately elevating outcomes for mothers and infants during critical situations.

Keywords
Low-Cost Model Task Trainer, Perimortem Cesarean Section, Resuscitative Hysterotomy, Clinical/Procedural Skills Training, Simulation

Educational Objectives

By the end of this activity, learners will be able to:

1. Demonstrate systematic, coordinated resuscitation based on Advanced Trauma Life Support principles.
2. Display leadership skills by demonstrating excellent teamwork, personnel management, situational awareness, and closed-loop communication.
3. Recognize maternal anatomical and physiological changes due to pregnancy, as well as their implications for resuscitation.
4. Demonstrate knowledge of special considerations in trauma resuscitation of a pregnant patient, such as estimating gestational age/viability, radiological imaging, Rho(D) immune globulin administration, fetal monitoring, and so on.
5. Demonstrate resuscitative hysterotomy.

Citation:
Chin HR, Ng WX. Low-cost, scalable simulations in obstetric trauma and resuscitative hysterotomy for emergency medicine residents. *MedEdPORTAL*. 2024;20:11452.
https://doi.org/10.15766/mep_2374-8265.11452

Introduction

Simulation-based learning plays a vital role in preparing health care providers to manage obstetric emergencies. High-acuity, low-occurrence events, such as severe obstetric trauma and maternal cardiac arrest, demand prompt action. However, due to these events' rare occurrence, emergency physicians often lack adequate experience in managing them. Simulation-based training bridges this gap by providing a safe environment to practice skills and improve confidence.

One specific procedure, resuscitative hysterotomy (RH), also known as perimortem cesarean section, is critical in maternal

Copyright © 2024 Chin and Ng. This is an open-access publication distributed under the terms of the Creative Commons Attribution license.

cardiac arrest. RH involves the emergency delivery of a fetus through an abdominal incision to optimize maternal resuscitation when maternal cardiac arrest occurs late in pregnancy or during labor. American Heart Association, American College of Obstetricians and Gynecologists, and Eastern Association for the Surgery of Trauma guidelines recommend initiating an RH within 4 minutes of maternal arrest and delivery of newborn within the fifth minute to optimize both fetal and maternal outcomes.[1-3] This time-critical, lifesaving procedure requires a high level of technical proficiency, coordination, and anatomical knowledge.

Commercial trainers for this procedure are often expensive, and previously described models in the literature[4-6] are often difficult to construct. Due to this, our residents lacked access to hands-on opportunities. Current training on the topic is limited to didactics and case-based discussions. To address this limitation, we developed a low-cost, easily replicable, and anatomically accurate model to provide our residents with hands-on practice opportunities. We aimed to assess the effectiveness of obstetric trauma simulation (incorporating the RH model) in improving skills and confidence for emergency physicians in training. This project was approved by the National Health Group (NHG) Domain Specific Review Board.

Methods

Development

A learning needs assessment, with the aim of identifying gaps in clinical knowledge and skills, was performed 3 months prior to the simulation session. We interviewed 10 residents (two from each year) and five core faculty of the NHG emergency medicine program on the topic and the current state of training. Residents expressed concerns about their lack of confidence with managing obstetric patients due to relatively low obstetric clinical caseloads. None of the residents had done or observed an RH, either in real life or in simulations. Faculty members mentioned that opportunities for practice and skills maintenance for practicing physicians were also limited due to the rarity of the procedure. No resuscitative hysterectomies had been done at the institution in the past 5 years at the time of writing.

To address the above needs, we wrote an obstetric trauma scenario with maternal arrest, necessitating RH. The aim was to allow residents to demonstrate the skills required to manage this scenario ("shows how" of Miller's pyramid[7]). The case scenario was written by a senior resident and a core faculty member in accordance with the above educational objectives. It depicted the trauma resuscitation of a young pregnant female, who eventually arrested and required RH in an emergency department resuscitation room. A test run of the scenario was conducted 1 week before the simulation session. No specific challenges were encountered.

Equipment/Environment

The simulation session was conducted at the Tan Tock Seng Hospital Simulation and Integrated Medical Training Advancement Centre (SIMTAC), simulating an emergency resuscitation bay. Equipment included one female mannikin with bruised gravid abdomen, one RH task trainer and one chest tube task trainer, monitoring devices, and other resuscitative equipment as detailed in Appendix A. We used low-cost and easily available material in constructing the RH task trainer, with the aim of increasing the replicability of the model. Both faculty members constructed five sets of RH task trainers about 1 week prior to the training session. The detailed steps in the construction of the RH task trainer are shown in Appendix B. The Table lists the materials and cost for the construction of the RH task trainer. The sum of the cost for the single-use materials was 17.35 Singapore dollars (as of September 2024, 1.00 Singapore dollar equaled approximately 0.77 US dollars).

Personnel

Two faculty and one technical assistant conducted the session. The first faculty member maintained oversight and controlled the flow of the scenario in the resuscitation room. The second faculty member assisted in the technical aspects of the simulation (e.g., changing vital signs, providing voice prompts, etc.) with a simulation center technical assistant in the control room. Faculty exchanged their roles after every cycle. No actors were required for this simulation.

The learners consisted of 30 NHG emergency residents spanning year 1 to year 5.

Implementation

The resuscitation bay was set up by our SIMTAC staff. Prereading[8] and groupings were disseminated to residents via email 1 week before the actual session. This provided the

Table. Resuscitative Hysterotomy Task Trainer Materials and Associated Cost

Item	Quantity	Price[a]
Aluminum tray	2	1.20
Baby doll (reusable)	1	13.00
40-cm beach play ball	1	1.70
Nippon paint spray	1	7.10
Adhesive tape and glue	1	2.20
Red foam craft	1	2.20
Cream felt craft	1	1.95
Kitchen cling wrap	1	1.00

[a]In Singapore dollars. As of September 2024, 1.00 Singapore dollar equaled approximately 0.77 US dollars.

Copyright © 2024 Chin and Ng. This is an open-access publication distributed under the terms of the Creative Commons Attribution license.

opportunity for the residents to acquire adequate textbook knowledge ahead of time.

Residents were split into five groups, with six participants per group. Each group had a balanced mix of residents, ranging from year 1 to year 5 in seniority. Five runs of the scenario were conducted. Each group participated in the scenario as a team. One resident took the role of team leader, while the rest took up roles as assigned by the leader.

This session was designed to simulate a resuscitation case in the emergency department. Learners were required to examine the mannikin; request investigations; specify the treatments, including medication dosages; and perform procedures such as CPR, intubation, and thoracostomy. Faculty provided them with information as the scenario unfolded. Appendix C describes the detailed flow of the simulation case scenario, including faculty actions, mannikin's description, and targeted learner's response. One resident from each group performed an RH on the task trainer under the guidance of a faculty members, while the others observed at the bedside. The scenario lasted approximately 20 minutes.

Debriefing

At the end of the scenario, a debrief to highlight pertinent considerations in resuscitating pregnant patients was conducted. Both faculty and peers provided feedback following the Pendleton feedback model.[9] Learners were given the opportunity to ask questions. The debrief took approximately 10-15 minutes per group.

Assessment

As this simulation program was designed as a practice session, we did not assess residents using a scoring matrix, instead choosing to focus on qualitative feedback. Faculty used observations of learners' actions to trigger discussions during the debriefings. After the end of the session, residents were invited to fill in an anonymous online questionnaire (Appendix D) assessing their perception on the relevance of training, rating of the task trainer, and their confidence level in dealing with a similar clinical scenario. One question had yes/no responses. One question asked respondents to rate the appropriateness of the simulation's complexity to learners' level of training. Responses to the rest of the questions were recorded using various 5-point Likert scales.

Results

Thirty emergency medicine residents ranging from year 1 to (final) year 5 participated in the obstetric trauma simulation. Twenty-three of the 30 participants (77%) responded to the questionnaire. A total of five runs of simulation scenarios were conducted in this session. All were conducted by the two faculty who designed the scenario. No specific challenges were identified during the session. Fifty-two percent of the participants had either performed, assisted, or observed a cesarean section previously. Ninety-six percent found the level of complexity appropriate. The Figure shows the responses to questionnaire items using 5-point Likert scales. Ninety-one percent of participants responded that the session increased their confidence in managing a similar clinical scenario (rated 4 or

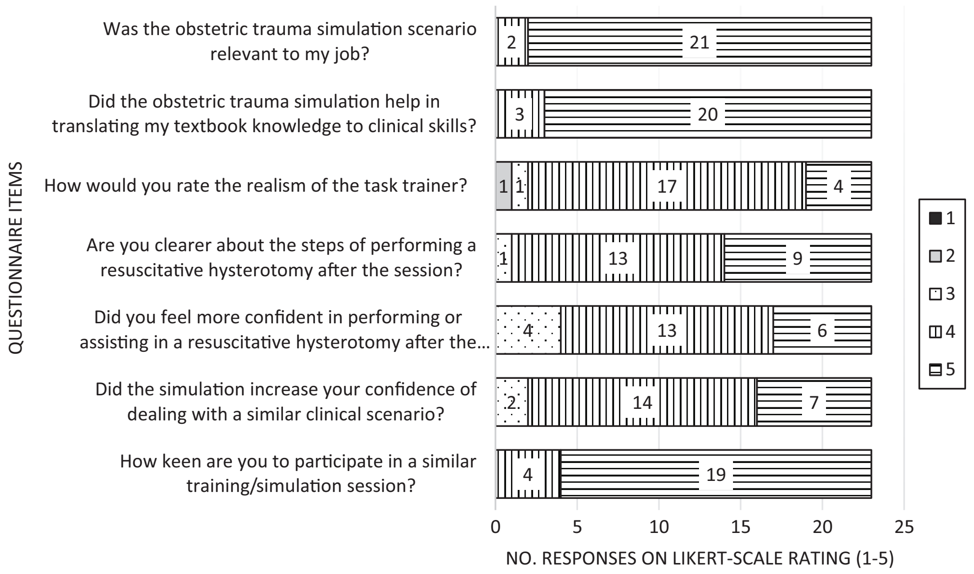

Figure. Questionnaire items and responses. Items were scored on various 5-point Likert scales, with 1 being the most negative response and 5 being the most positive.

Copyright © 2024 Chin and Ng. This is an open-access publication distributed under the terms of the Creative Commons Attribution license.

5 on the Likert scale). Ninety-one percent of participants felt that the task trainer was realistic (rated 4 or 5 on the Likert scale).

Discussion

Residents found the session relevant and tailored to an appropriate level. They felt that the session helped to translate textbook knowledge to clinical skills. They reported increased confidence after the session, which is consistent with numerous studies[10-12] that have shown that simulation-based learning improves health care providers' knowledge, technical skills, and confidence in managing obstetric emergencies.

Despite its cost-effectiveness and ability to mimic actual anatomical layers, the task trainer fell short in replicating the textures of different human soft tissue layers. We intend to improve the realism of future models by experimenting with different materials to better simulate each anatomical layer while still controlling costs. We believe that this task trainer could be adapted for the use of surgical residents, such as obstetrics/gynecology and general surgery.

The session's ability to reflect the educational impact might be limited due to the varied seniority levels of participating learners, from year 1 to year 5. This variation in seniority could lead to a range of responses on survey items. The anonymous nature of the survey limited our ability to analyze responses by seniority. Future studies should consider methodologies that collect demographic information while preserving anonymity and the voluntary nature of participation. Such approaches can help tailor educational activities more effectively to the specific needs of different learner groups.

One notable limitation of this session was the absence of an assessment rubric, which restricted our ability to measure learner competency accurately. We hope to incorporate a robust assessment framework in future sessions. Regular sessions are also planned to reinforce cognitive schemas and muscle memory, which are crucial for managing obstetric emergencies.

We recognize the emotional challenges posed by managing obstetric emergencies, even in a simulated setting. Moving forward, we hope to integrate trauma-informed debriefing practices[13,14] into future sessions to address potential learner distress.

Obstetric trauma simulation and RH are important aspects of emergency obstetric care. Through simulation-based exercises, health care providers can acquire necessary skills, improve teamwork and communication, and develop the ability to manage complex obstetric emergencies. The use of simulation allows for a safe and controlled environment in which to practice and learn, ultimately leading to improved patient outcomes. By embracing this innovative educational approach, health care professionals can better prepare for rare and challenging obstetric emergencies, ensuring that mothers and babies receive optimal care during critical situations.

Appendices

A. List of Required Equipment.docx

B. Resuscitative Hysterotomy Task Trainer Construction.docx

C. Simulation Case.docx

D. Questionnaire.docx

All appendices are peer reviewed as integral parts of the Original Publication.

Hao Ren Chin, MBBS, MRCEM: Associate Consultant, Department of Emergency Medicine, Tan Tock Seng Hospital; ORCID: https://orcid.org/0000-0001-8867-4023

Wei Xiang Ng, MBBS, MMed (Emergency): Consultant, Department of Emergency Medicine, Tan Tock Seng Hospital

Disclosures

None to report.

Funding/Support

None to report.

Ethical Approval

The National Health Group Domain Specific Review Board approved this project.

References

1. Vanden Hoek TL, Morrison LJ, Shuster M, et al. Part 12: cardiac arrest in special situations: 2010 American Heart Association guidelines for cardiopulmonary resuscitation and emergency cardiovascular care. *Circulation*. 2010;122(18)(suppl 3): S829-S861. https://doi.org/10.1161/CIRCULATIONAHA.110.971069
2. Nolan JP, Soar J, Zideman DA, et al; ERC Guidelines Writing Group. European Resuscitation Council guidelines for resuscitation 2010: section 1. executive summary. *Resuscitation*. 2010;81(10):1219-1276. https://doi.org/10.1016/j.resuscitation.2010.08.021

Copyright © 2024 Chin and Ng. This is an open-access publication distributed under the terms of the Creative Commons Attribution license.

3. Einav S, Kaufman N, Sela HY. Maternal cardiac arrest and perimortem caesarean delivery: evidence or expert-based? *Resuscitation*. 2012;83(10):1191-1200. https://doi.org/10.1016/j.resuscitation.2012.05.005
4. Palm KH, Lei C, Walsh R, Heimiller J, Sikon J. An inexpensive, high-fidelity resuscitative hysterotomy (RH) model with hemorrhage capability. *Cureus*. 2022;14(6):e25582. https://doi.org/10.7759/cureus.25582
5. Lau L, Papanagnou D, Smith E, Waters C, Teixeira E, Zhang XC. A novel biosimulation task trainer for the deliberate practice of resuscitative hysterotomy. *Adv Simul (Lond)*. 2018;3:19. https://doi.org/10.1186/s41077-018-0078-1
6. Sampson CS, Renz NR, Wagner JC. An inexpensive and novel model for perimortem cesarean section. *Simul Healthc*. 2013; 8(1):49-51. https://doi.org/10.1097/SIH.0b013e318271489c
7. Miller GE. The assessment of clinical skills/competence/performance. *Acad Med.* 1990;65(9):S63-S67. https://doi.org/10.1097/00001888-199009000-00045
8. Smith KA, Bryce S. Management of the pregnant trauma patient in the emergency department. *Emerg Med Pract*. 2020;22(suppl 10):1-36.
9. Pendleton D, Schofield T, Tate P, Havelock P. *The Consultation: An Approach to Learning and Teaching.* Oxford University Press; 1984.
10. Fisher N, Eisen LA, Bayya JV, et al. Improved performance of maternal-fetal medicine staff after maternal cardiac arrest simulation-based training. *Am J Obstet Gynecol*. 2011;205(3): 239.e1-239.e5. https://doi.org/10.1016/j.ajog.2011.06.012
11. O'Dea M, Murphy D, Dubrowski A, Rogers P. Optimizing perimortem cesarean section outcomes using simulation: a technical report. *Cureus*. 2020;12(9):e10588. https://doi.org/10.7759/cureus.10588
12. Hunt EA, Shilkofski NA, Stavroudis TA, Nelson KL. Simulation: translation to improved team performance. *Anesthesiol Clin*. 2007;25(2):301-319. https://doi.org/10.1016/j.anclin.2007.03.004
13. Harder N, Lemoine J, Chernomas W, Osachuk T. Developing a trauma-informed psychologically safe debriefing framework for emotionally stressful simulation events. *Clin Simul Nurs*. 2021;51:1-9. https://doi.org/10.1016/j.ecns.2020.11.007
14. Fallot RD, Harris M. *Trauma-Informed Services: A Self-Assessment and Planning Protocol.* Community Connections; 2006.

Received: December 9, 2023
Accepted: June 6, 2024
Published: October 3, 2024

Copyright © 2024 Chin and Ng. This is an open-access publication distributed under the terms of the Creative Commons Attribution license.
